# The role of physical and cognitive function in performance of activities of daily living in patients with mild-to-moderate Alzheimer’s disease – a cross-sectional study

**DOI:** 10.1186/s12877-020-01926-9

**Published:** 2020-11-27

**Authors:** Frederikke K Clemmensen, Kristine Hoffmann, Volkert Siersma, Nanna Sobol, Nina Beyer, Birgitte B Andersen, Asmus Vogel, Annette Lolk, Hanne Gottrup, Peter Høgh, Gunhild Waldemar, Steen G Hasselbalch, Kristian S Frederiksen

**Affiliations:** 1 Danish Dementia Research Centre, Department of Neurology, Rigshospitalet, Copenhagen University Hospital, Blegdamsvej 9 – section 8025, 2100 Copenhagen, Denmark; 2 Regional Dementia Research Centre, Department of Neurology, Zealand University Hospital, Roskilde, Denmark; 3 Research Unit for General Practice and Section of General Practice, Department of public health, University of Copenhagen, Copenhagen, Denmark; 4 Department of Physical and Occupational Therapy and Institute of Sports Medicine, Bispebjerg Hospital, University of Copenhagen, Copenhagen, Denmark; 5 Dementia Clinic, Odense University Hospital, Odense, Denmark; 6 Dementia Clinic, Aarhus University Hospital, Aarhus, Denmark

**Keywords:** Alzheimer’s disease, Dementia, Physical function, Aerobic exercise, Activity of daily living, Cognition, Executive functions

## Abstract

**Background:**

Several factors may play a role in the ability of patients with Alzheimer’s disease to perform activities of daily living (ADL). The aim of this study was to examine the impact of different aspects of physical performance and cognitive functions on ADL in patients suffering from mild-to-moderate Alzheimer’s disease.

**Methods:**

We conducted secondary analyses on cross-sectional baseline data from the randomized controlled multicentre study “Preserving quality of life, physical health and functional ability in Alzheimer’s Disease: The effect of physical exercise” (ADEX). In total, 185 AD patients (76 women and 109 men), with a mean age on 70,4 years, were included. Data from physical performance tests (Astrand cycle test, Timed up & Go (TUG), Sit to Stand test (STS)) and cognitive tests (Mini Mental Status Examination (MMSE), Symbol Digit Modalities Test (SDMT), Stroop Color and Word test (Stroop)) were used. Their associations with ADL, measured on the ADCS-ADL scale was assessed in multivariable regression analyses.

**Results:**

SDMT and MMSE had significant, moderate correlations with total ADL (SDMT: *r* = 0.33, MMSE: *r* = 0.42) and instrumental ADL (SDMT: *r* = 0.31, MMSE: *r* = 0.42), but not with basic ADL. Adjusting for age and sex, the associations between SDMT and MMSE to total ADL and instrumental ADL persisted. No significant associations were found between Astrand, TUG, STS or Stroop and total ADL, basic ADL or instrumental ADL.

**Conclusion:**

Total ADL and instrumental ADL are associated with cognitive functions, including executive function. No significant association between examined physical performance parameters and ADL functions was observed, and consequently does not support an impact of physical function on ADL functions in patients with mild-to-moderate Alzheimer’s disease and relatively well-preserved physical function. Strategies aimed to improve cognition may be better suited to improve ADL function in patients with mild-to-moderate Alzheimer’s disease.

**Trial registration:**

NCT01681602. Registered 10 September 2012, retrospectively registered.

## Background

Alzheimer’s disease (AD) is a neurodegenerative disease with early and prominent memory impairment, and decline in the ability to independently carry out activities of daily living (ADL) [1–3]. ADL functions may be divided into basic ADL (BADL) (e.g. eating and personal hygiene) and instrumental ADL (IADL) (e.g. using a telephone and shopping) [4]. IADL have been found to decline in early stages and may be more related to cognitive abilities, while BADL decline in more advanced stages and may be less dependent on cognitive functioning [5].

Several patient-related factors such as cognitive function, neuropsychiatric symptoms and disease duration may have deleterious impact on ADL functions in AD [6–8], patients with Mild Cognitive Impairment (MCI) [9] and in older adults receiving domiciliary care [10]. Moreover, physical performance of the patients (e.g. balance, strength and aerobic capacity) is likely to play a role. For example, the Timed Up and Go (TUG), a measure of basic mobility, has been shown to predict future functional dependency in a large cohort [11]. Environment-related factor may also affect the ability of patients to perform ADL including spatial layout [12], lighting [13], design of dining rooms [14] and access to outdoor areas [15]. This clearly indicates that the performance of ADL is a complex process reliant on both cognitive and physical performance of the patient as well as the environment inhabited by the patient. A further example of the intricacies of mobility, ADL and cognition was found in a study looking at strategies employed by patients with dementia to perform “sit to stand”. Patients with dementia on average employed more different strategies than cognitively unimpaired [16]. For patients with dementia “Sit to stand” remains an important function to be able to perform in order to maintain ADL [17].

With regards to the relationship between cognition and ADL [1, 2, 18], deterioration of executive function has been specifically associated with poorer performance on ADL functions in patients with Alzheimer’s disease [19–22]. Physical exercise has been found to improve the ability to perform ADL functions in both AD patients [23–26] and in community-dwelling older adults [27–30], although results are not consistent across studies in either populations [31, 32].

Other interventions have also shown to be able to improve ADL in patients with MCI and dementia. For example, a combination of cognitive-physical training has shown beneficial in both patients with dementia and MCI [33]. The approach of combined cognitive-motor exercise, i.e. training both cognitive abilities and motor skills/physical performance has been shown to possibly improve ADL functions in MCI patients [34], and may be superior to physical exercise alone [35]. Further, cognitive rehabilitation interventions may also improve ADL, as well as cognitive training. These interventions usually involve repeated sessions where either a specific ADL function (e.g. making coffee) is trained (cognitive rehabilitation) or where underlying cognitive functions which may sub serve ADL functions are trained. Both approaches has been shown to possibly improve ADL functions as well as cognitive functions in patients with dementia [36–38]. A recent meta-analysis found an impact of multicomponent training interventions (aerobic, strength, postural and balance exercise) on ADL, but not on cognitive function and physical fitness, reinforcing the importance of analyzing subdomain changes through more specific outcomes, also considering different stages of dementia [39].

Preservation of executive function, rather than memory, has been linked to slower decline in ADL in AD patients [40] and further, executive function, but not memory, has been found to correlate with IADL in non-demented older adults [41] and has been found to explain more of the variance in IADL [42]. The abovementioned suggests that executive function may be more important than memory for performing ADL functions. Numerous studies have shown that aerobic fitness, strength and balance can be improved in patients with dementia [43]. Physical exercise has also been found to improve cognitive function in patients with MCI and dementia [44, 45]. Further, especially executive functions may be specifically positively affected by physical exercise in AD [46–49] as well as in healthy older persons [50–52].

A better understanding of the individual contributions of different cognitive functions and the impact of physical performance would potentially enable targeted interventions aimed at improving ADL functions. As stated above many studies have investigated the relationship between physical performance, cognition and ADL, in isolation. However, how all three variables are related still remains unclear. An exception is a rather recent cross-sectional study in which it was reported that the relationship between aerobic fitness and ADL function in AD patients was mediated by global cognition [53].

The objectives of the present study were to examine the possible association between physical performance, cognition, and ADL functions. We hypothesised that physical performance and cognition, respectively, were associated with ADL functions. Further we hypothesised that IADL is more cognitively taxing than BADL, and therefore less dependent of physical function and the converse regarding IADL. Further, we wished to assess the impact of physical performance and cognitive functions on ADL, and to examine the possible mediating role of cognitive function with regards to the relationship between physical performance and ADL function.

## Methods

### Study design

**Fig. 1:**
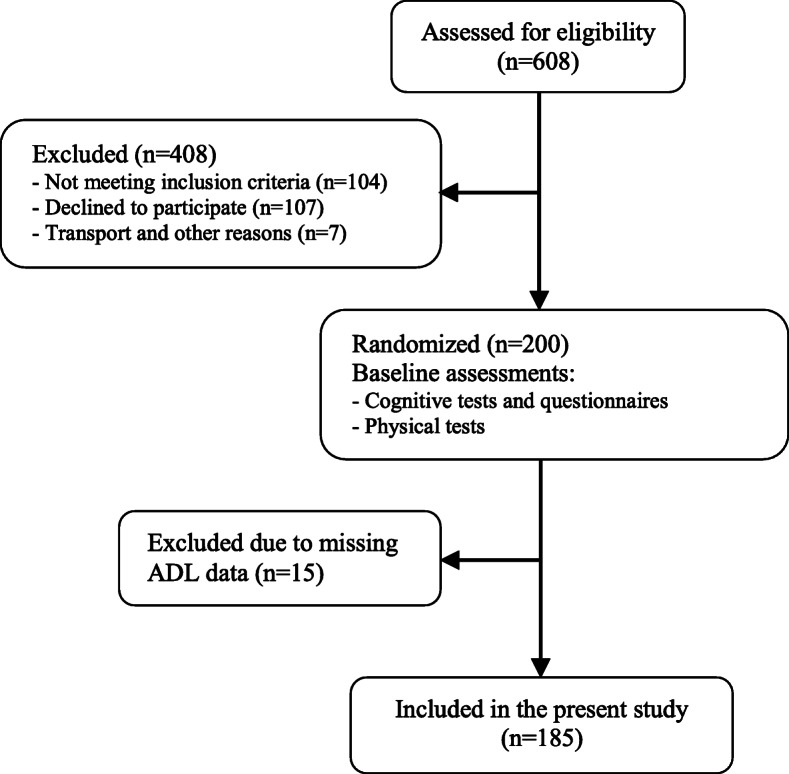
Flow chart of the ADEX study

This is a secondary analysis of cross-sectional data from a previously conducted randomized controlled trial using baseline data from patients enrolled in the ADEX study (Preserving quality of life, physical health and functional ability in Alzheimer’s disease: The effect of physical exercise study). The ADEX study was a multicentre single-blinded study, conducted between January 2012 and June 2014, investigating the impact of a 16-week aerobic exercise intervention on symptoms of AD [45, 54]. Flow chart of the study is presented in Fig. 1. The ADEX study was approved by the Danish National Committee on Biomedical Research Ethics (H-3-2011-128) and written informed consent was obtained from each participant prior to enrolment.

### Participants

A total of 200 community-dwelling patients with mild-to-moderate AD were included in the ADEX study, all meeting criteria for probable AD according to the Alzheimer’s criteria by the *National Institute of Neurological and Communicative Disorders and Stroke and the Alzheimer’s Disease and Related Disorders* (NINDS-ADRDA), McKhann et al., 1984. All participants were diagnosed by a physician who was a specialist in either geriatric medicine, psychiatry or neurology. As a part of the diagnostic process, all subjects underwent as a minimum formal cognitive testing, ADL assessment, blood samples and structural brain scans (CT or MRI). Patients were recruited from and followed in 8 memory clinics in Denmark. Details on objectives and design including inclusion and exclusion criteria have previously been described in detail [54]. For the present study, 185 of the 200 participants (92.5%) had sufficient baseline data for variables of interest and were included in the present study.

### Cognitive assessments

Cognitive assessments were performed in a quiet environment without interruptions. The Mini-Mental State Examination (MMSE) was used to assess global cognitive function [55]. Processing speed and attention were assessed by the Symbol Digit Modalities Test (SDMT). For the present study, the number of correct responses within a 120-s response time was used [56]. The Stroop Colour and Word Test was applied to assess executive control and inhibition [57]. In this study, the number of words the participant read in 45 s (maximum score of 100) in the colour word test (incongruent version) was used for analysis.

### Assessments of physical performance

To assess aerobic fitness, the 6-min Astrand Cycle Ergometer test (Monark Ergomedic 839E, Monark Exercise AB, Sweden) was used. The estimated maximum oxygen uptake (VO_2_max (ml/kg/min)) based on workload and average heart rate during the last minute of the test, corrected for age and body weight [58], was calculated. The Timed Up & Go test (TUG) was used to assess basic mobility [59]. The 30 s chair stand test, sit to stand (STS) was used to assess strength and endurance in the lower extremities [60].

### Assessment of activities of daily living

ADL was assessed by the Alzheimer’s Disease Co-operative Study - ADL (ADCS-ADL) scale [4]. The ADCS-ADL scale assesses 23 ADL items and is administered to the caregiver as an interview. The scale comprises two components; basic ADL (BADL) consisting of six items such as self-feeding, personal hygiene, and dressing, and instrumental ADL (IADL) consisting of 17 items such as using the telephone, reading books or magazines, managing finances, leisure activities and household chores. The BADL score ranges from 0 to 22 and the IADL from 0 to 56. Together BADL and IADL comprise total ADL with the range 0–78. The higher score indicates less impairment.

### Statistical analysis

Unadjusted associations between total ADL, BADL and IADL, and measures of physical performance (Astrand test, TUG, STS) and cognitive function (MMSE; Stroop and SDMT) were assessed by Pearson’s correlation coefficient. Using multivariable linear regression models the beforementioned relationships were examined (adjusted for age and sex) by the corresponding β-coefficients where the measures of physical performance and cognitive function where entered individually. In order to assess a potential mediating effect of cognition on the association between physical performance and ADL, the regressions for the measures of physical performance were additionally adjusted for the measures of cognitive functions (MMSE, Stroop and SDMT). A small number of patients had missing data for Stroop, SDMT and Astrand, which were handled with listwise deletion. Statistical analyses were performed using SAS v9.4 and R v3.5.1. Statistical significance was accepted at *p* < 0.05.

## Results

Baseline characteristics for all 185 participants included in the study are presented in Table 1. The average age was 70.4 years, with a mean MMSE score of 24.6. Seventy-six (41%) participants were women. The mean total ADL was 64.2, indicating mildly affected ADL. Participants were in general in good health with very few comorbidities (see Table 1), below that of average patients with AD [61].

**Table 1:** Demographics

Characteristics	N 185Mean (SD)185
*Age, years (SD)*	70.4 (7.4)
*Gender (women), n (%)*	76 (41%)
***Comorbidities, n (%)***	
*Hypertension*	75 (40,5)
*Diabetes*	18 (9,7)
*Hypercholesterolemia*	68 (36,8)
*Apoplexia*	3 (1,6)
*Acute myocardial infarction*	3 (1,6)
*Walking aid*	0 (%)
***Cognitive performance, mean (SD)***	
*MMSE*	24.6 (3.0)
*SDMT*	27.3 (14.3)
*Stroop*	18.4 (9.8)
***Physical performance, mean (SD)***	
*Astrand (VO*_*2*_*max)*	25.9 (8.2)
*TUG (sec)*	6.6 (1.5)
*STS (number in 30 s)*	14.6 (3.9)
***Activities of Daily Living, mean (SD)***	
*ADL*	64.2 (9.3)
*BADL*	21.1 (1.6)
*IADL*	43.0 (8.8)

*MMSE* Mini Mental State Examination, *SDMT* Symbol Digit Modalities Test, *ADL* Activities of Daily Living, *BADL* Basic ADL, *IADL* Instrumental ADL, *TUG* Timed Up & Go, *STS* Sit to Stand

Pearson correlation coefficients (PCC) are presented in Table 2. SDMT was significantly and positively correlated with total ADL (*r* = 0.33, *P* < 0.05) and IADL (*r* = 0.31, *P* < 0.05), but not with BADL. Moreover, MMSE was positively and significantly correlated with total ADL (*r* = 0.42, *P* < 0.05) and IADL (*r* = 0.42, *P* < 0.05), but not with BADL. None of the physical parameters nor Stroop were correlated with ADL.

**Table 2:** Correlation and Regression coefficients

	PCC	Regression coefficients (RC)			RC adjusted for cognitive function		
	*r*	β	95% CI	*p*-value	β	95% CI	*p*-value
*Total ADL*							
*Astrand*	0	0.06	− 0.12 - 0.35	0.50	0.01	− 0.15 - 0.18	0.88
*TUG*	−0.03	− 0.05	− 0.97 - 0.87	0.92	0.35	− 0.52 - 1.22	0.43
*STS*	0.07	0.20	−0.14 - 0.54	0.24	0.05	−0.28 - 0.37	0.77
*SDMT*	0.33*	0.14*	0.05–0.23	0.0019			
*Stroop*	0.08	0.09	−0.04 - 0.22	0.20			
*MMSE*	0.42*	1.19*	0.81–1.58	< 0.00001			
*BADL*							
*Astrand*	0.1	0.01	−0.02 - 0.04	0.51	0.01	−0.02 - 0.04	0.43
*TUG*	−0.06	−0.03	− 0.19 - 0.12	0.68	− 0.05	−0.22 - 0.12	0.56
*STS*	0.06	0.02	−0.03 - 0.08	0.40	0.02	−0.05 - 0.08	0.60
*SDMT*	0.22	0.01	−0.01 - 0.02	0.28			
*Stroop*	0.03	−0.01	−0.03 - 0.02	0.64			
*MMSE*	0.18	0.05	−0.02 - 0.11	0.20			
*IADL*							
*Astrand*	0	0.05	−0.13 - 0.22	0.58	0.01	−0.15 - 0.16	0.94
*TUG*	−0.03	−0.01	− 0.88 - 0.85	0.98	0.40	−0.40 - 1.21	0.32
*STS*	0.07	0.17	−0.14 - 0.49	0.29	0.04	−0.26 - 0.34	0.78
*SDMT*	0.31*	0.13*	0.05–0.21	0.0047			
*Stroop*	0.09	0.09	−0.03 - 0.21	0.14			
*MMSE*	0.42*	1.13*	0.78–1.47	< 0.00001			

*P < 0.05. All regression coefficients are adjusted for sex and age. *PCC* Pearson Correlation Coefficient, *ADL* Activities of daily living, *BADL* Basic ADL, *IADL* Instrumental ADL, *TUG* Timed up & go, *STS* Sit to stand, *MMSE* Mini mental state examination, *SDMT* SYMBOL Digit Modalities Test, *RC* regression coefficients

The corresponding β-coefficients from multivariable linear regression models, adjusted for age and sex, are presented in Table 2. Results show that SDMT was associated with total ADL (β = 0.14, *p* = 0.0019) and IADL (β = 0.13, *p* = 0.0047). Similarly, MMSE was associated with total ADL (β = 1.19, *p* < 0.00001) and IADL (β = 1.13, *p* < 0.00001). Concerning BADL, no significant associations with physical nor cognitive parameters were found. No significant associations were found between Astrand, TUG, STS or Stroop and ADL, BADL or IADL.

Since no association between physical performance parameters and ADL functions were found, a mediating effect of cognition was not excepted. However, the regressions for physical performance parameters adjusted for the measures of cognitive functions are presented in Table 2. These results show a minor change for total ADL and IADL, where the β-coefficient for Astrand and STS decreases, while the β-coefficient for TUG increases. No change was observed in BADL.

## Discussion

In the present study, we add to the limited research on how physical performance and cognitive subdomains impact on ADL in patients with mild-to-moderate AD.

We found that cognition and ADL were significantly and positively correlated. Specifically, with regards to cognitive domains we found that processing speed and attention correlated significantly with IADL but not BADL. The findings support our hypothesis that cognitive function is of importance for ADL functions and especially for more complex ADL functions captured by IADLs [3]. Measures of physical performance were not associated with ADL. To our knowledge this is the largest study to date examining the relationship between ADL, aerobic function and cognition.

In general, a significant moderate correlation was observed between general cognitive function and the instrumental subscale of ADL, while no correlation was found to basic ADL, suggesting that IADL is more reliant on cognition. When examining the relationship in more detail, processing speed and attention were found to be associated with total ADL and IADL, but not BADL, mirroring the findings for general cognitive function. As peviously stated, the findings may indicate that those complex activities comprising IADL are most reliant on cognition. Supporting this finding, IADL has been found to be more strongly associated with executive function than BADL in patients with mild-to-moderate AD [21, 62]. Moreover, in older adults without dementia deterioration in executive function is independently correlated with the rate of change in IADL, and may be more deleterious, even in the presence of memory deficits [41]. However, it should be kept in mind that the participants included in the present study suffered from mild-to-moderate AD. Therefore, these findings may also reflect that IADL was primarily affected, as compared to BADL which is typically affected in later stages of dementia [5]. This is also in line with the findings in the present study.

Another important finding is the lack of significant association between ADL functions and physical performance i.e. aerobic fitness, measure of mobility, and strength and endurance of lower extremities, which is not consistent with the findings of a recent meta-analysis establishing an association between TUG and STS and worsening in ADL in populations of older adults [63]. Our results may indicate that physical performance does not have a significant impact on ADL functions in our sample of AD patients in the mild to moderate stage. Considering that participants in our study were likely more physically fit compared to AD patients in general, a ceiling effect on physical performance measures is also possible. Furthermore, the participants were also relatively unimpaired in ADL function, which together may have masked a possible relationship. Nevertheless, our findings are in accordance with a similar recent study, which to our knowledge is the first study to examine the impact of aerobic fitness on ADL, in which no direct effect of aerobic fitness on ADL functions was found [53]. Yu et al. further found that the relationship between aerobic fitness and ADL was mediated by a number of factors including global cognition. Although we did not find a significant mediating effect of cognition between physical performance and ADL, we did observe a decrease in the β-coefficients for the physical performance parameters concerning ADL and IADL (Table 2), which may nevertheless indicate some mediating effect of cognition.

Our findings add to the relatively limited research on the role of aerobic and physical performance on ADL in patients with AD. The findings provide important input for future design of interventions aimed at improving ADL. Our findings of an association between cognitive function, and specifically a measure of procession speed and mental speed, indicate that interventions in patients with mild to moderate AD focusing on improving these cognitive functions may be more beneficial for improving ADL. The fact that we did not find an association between ADL and aerobic performance would seem to indicate that in mild to moderate AD, interventions to improve ADL functions may be less effective, if targeting aerobic function. It may be speculated, based on our findings that in more advanced patients, interventions improving aerobic and physical performance may improve ADL through an effect on BADL. In order to further investigate the impact of physical performance on ADL, especially considering recent research regarding effects of cognitive-motor interventions on ADL [35, 39], it would be interesting to investigate the association between domain specific cognitive decline, decline in physical performance and in ADL functions in a cognitive-motor intervention study on AD patients.

We acknowledge that this study has some limitations. The study is a cross-sectional study excluding an analysis of a causal relationship. Analyses of intervention is of interest in order to investigate how physical performance, cognition and ADL benefit from physical exercise [43, 45]. However, this study focused on the mutual relationship between the three parameters and not the impact of the intervention on this relationship. The study included a selected group of patients with relative mild cognitive symptoms and relatively physically fit, prior to an intervention with physical exercise, potentially excluding patients with greater impairment in ADL. Therefore, selection bias is a risk, and the results may not be generalizable to all patients with AD, e.g. patients with more impairment in ADL, less physically fit patients, or patients with more comorbidities. We did not control for comorbidities in the statistical analysis, as our population had relatively few comorbidities, but we cannot exclude that these did not affect our results, which we acknowledge as a limitation. Further, we acknowledge that the low variability in our sample group concerning physical performance, could potentially underestimate the associations. This is a secondary analysis of data from a previously conducted intervention trial, and it cannot be ruled out that the study was underpowered. We acknowledge that several physical barriers affect ADL functions, and that impairment in ADL is most likely caused by multiple factors. However, it is not possible to take all such factors into account and the main focus in this study was on selected physical and cognitive tests. The ADCS-ADL scale assesses different aspects of everyday living but may not capture all relevant aspects of ADL. It is a strength that our large group of patients are well characterized and have been assessed using a diverse number of scales and physical performance measures.

## Conclusion

In conclusion, this study found a significant and positive association between cognition, including processing speed and attention and total ADL and IADL functions in patients with mild-to-moderate AD. No association was found between cognition and BADL, suggesting that BADL is less cognitively demanding and less reliant on cognitive function. Although our findings do not support physical function as being important for performance of ADL functions in patients with mild-to-moderate AD and with relatively well-preserved physical function, it cannot be interpreted as if such an association does not exist in persons with more severe dementia or more severe ADL impairment. Our study could imply that interventions targeting cognition may also have a positive effect on instrumental ADL in patients with mild-to-moderate AD.

## Data Availability

The datasets used and analysed in this study are available from the corresponding author on reasonable request.

## Abbreviations

AD: Alzheimer’s Disease
ADCS-ADL: Alzheimer’s Disease Co-operative Study – Activities of Daily Living
ADL: Activities of Daily Living
BADL: Basic Activities of Daily Living
IADL: Instrumental Activities of Daily Living
MCI: Mild Cognitive Impairment
MMSE: Mini Mental Status Examination
NINDS-ADRDA: National Institute of Neurological and Communicative Disorders and Stroke and the Alzheimer’s Disease and Related Disorders Association
PCC: Pearson correlation coefficients
SDMT: Symbol Digit Modalities Test
Stroop: Stroop Colour and Word test
STS: Sit to Stand test
TUG: Timed up & Go
